# TinyML pipeline for efficient crack classification in UAV-based structural health inspections

**DOI:** 10.1038/s41598-026-43534-4

**Published:** 2026-03-12

**Authors:** Yuxuan Zhang, Arne Nürnberg, Luciano Sebastian Martinez Rau, Quynh Nguyen Phuong Vu, Yuchen Lu, Bengt Oelmann, Sebastian Bader

**Affiliations:** 1 College of Intelligent Science and Engineering, Beijing University of Agriculture, Beijing, China; 2 Department of Computer and Electrical Engineering, Mid Sweden University, Sundsvall, Sweden; 3 Instituto de Investigación en Señales, Sistemas e Inteligencia Computacional, sinc(i), FICH-UNL/CONICET, Santa Fe, Argentina; 4 Yantai Research Institute, Harbin Engineering University, Yantai, China

**Keywords:** TinyML, Convolutional neural networks, Structure health monitoring, Crack classification, Embedded systems, Model compression, Engineering, Mathematics and computing

## Abstract

Structural health monitoring (SHM) of civil, aerospace, and energy infrastructure increasingly relies on UAVs with vision sensors for efficient inspections. Crack classification is a central task, yet cloud-based inference introduces bandwidth, power, connectivity, and privacy challenges that limit its practicality. This study presents a fully self-contained Tiny Machine Learning (TinyML) pipeline for onboard crack classification on a milliwatt-level STM32H7 microcontroller. Using MobileNetV1x0.25 as the baseline, we systematically evaluate the full measurement pipeline, including image capture, preprocessing, and inference on a low-power embedded system. Two preprocessing strategies, a handcrafted sequence (grayscale, contrast, denoise, median, binarization) and a greedy algorithm-based composite method, are compared. Four compression techniques, namely post-training quantization (PTQ), quantization-aware training (QAT), pruning, and weight clustering, are assessed individually and in combination. The optimized pipeline achieves an F1-score of 0.938, an improvement of 11.4% over state-of-the-art deployments. At the same time, it requires only 2.9 MB RAM and 309 KB flash, with an end-to-end latency of 461.6 ms and an energy cost of 623.16 mJ per inference. On a DJI Mini 4 Pro UAV, continuous operation reduces flight time by just 1.31 minutes (4%), compared to 8 minutes (24%) when using Jetson-based platforms. Overall, this work delivers a reproducible benchmark for UAV-based SHM, demonstrating a practical balance of accuracy, resource efficiency, and energy consumption, and advancing the feasibility of on-device crack classification in highly resource-constrained environments.

## Introduction

Structural Health Monitoring (SHM) is essential for aging civil, aerospace, and energy infrastructures, such as bridge SHM, enabling early detection of degradation to prevent failures and reduce life-cycle costs^1–4^. Traditional SHM systems mainly use time-series data from contact-based sensors, such as strain gauges, accelerometers, or acoustic emission arrays, to assess structural integrity^5–7^. Although effective in many scenarios, these approaches have two major drawbacks: they primarily detect dynamic effects caused by damage, which often leads to late-stage detection, and they involve high installation costs, especially when extensive sensor coverage is needed across the structure. To overcome these limitations, visual inspection has emerged as a complementary technique that enables early damage identification without the need for costly and dense sensor installations. However, manual surveys are labor-intensive, subjective, and can require disruptive access measures like lane closures or scaffolding^8^. This has driven the development of autonomous vision-based systems, offering high-resolution, objective, and repeatable assessments with greater efficiency^9–12^. Among these, UAVs equipped with vision modules have emerged as a promising platform for automated structural inspections, forming the basis of this study.

Among autonomous vision-based SHM systems, crack classification is widely regarded as a canonical benchmark task^13^. The prevailing workflow allows a UAV to acquire images in situ and to upload the raw data to a remote server or cloud cluster, delegating inference to a powerful deep-learning model^14^. Although this “edge acquisition–cloud inference” paradigm capitalizes on virtually unlimited compute resources, it simultaneously exposes critical weaknesses: (i) continuous high-bandwidth streaming of image data drains the limited battery budget and shortens flight time; (ii) intermittent or degraded connectivity jeopardizes mission reliability for safety-critical assets; and (iii) disseminating infrastructure images to external servers raises security and privacy concerns for owners and regulators^15–17^. Consequently, there is a need for on-board execution of crack-classification models on low-power, resource-constrained hardware.

Early efforts to run deep-learning inference directly on UAVs have relied on edge computers such as the Raspberry Pi, NVIDIA Jetson series, or Qualcomm Snapdragon 850^8^. While these single-board platforms can accommodate midsize networks, their continuous power draw of the order of several watts has considerable effects on the flight time of lightweight UAVs. Tiny Machine Learning (TinyML) on microcontroller units (MCUs) has recently enabled neural network execution at milliwatt-level power budgets^18–20^. Nonetheless, the limited on-chip RAM and flash memories impose stringent constraints on both model parameters and intermediate activations, necessitating model compression. In^14^, 14 lightweight convolutional neural networks have been benchmarked demonstrating that seven of them can successfully conduct crack classification on a low-power MCU. While MobileNetV1x0.25 (MBNV1x0.25) has been identified to provide a good trade-off between accuracy and resource requirements, the reported accuracy of the compressed model was limited to 76%, which is low for practical applications.

Model accuracy in edge-based vision tasks is not solely determined by network architecture but is also strongly affected by other stages of the processing pipeline, particularly image preprocessing and model compression. In TinyML deployments, preprocessing enhances discriminative visual cues while shaping the input statistics that quantized models must accommodate. Prior studies have shown that appropriate preprocessing can improve classification performance by emphasizing task-relevant structures and suppressing background noise^21–23^. However, how preprocessing operations should be composed remains unclear. A common practice is to adopt fixed, sequential pipelines based on heuristic design, implicitly assuming that each added operation contributes positively and independently. In contrast, greedy, performance-driven strategies explicitly evaluate the marginal benefit of each preprocessing step under deployment constraints to construct compact and effective pipelines. To date, the motivation, effectiveness, and trade-offs between these two design paradigms, sequential versus greedy, have not been systematically investigated for autonomous crack classification on resource-constrained MCUs. Similarly, model compression choices, particularly quantization strategies, critically affect the accuracy–efficiency trade-off in edge inference^24,25^. While methods such as pruning, and weight clustering have been studied individually, their interaction with different preprocessing strategies remains poorly understood. As a result, the combined impact of preprocessing design and model compression on end-to-end TinyML-based UAV crack inspection pipelines is still largely unexplored.

To address this challenge, we propose an end-to-end crack classification inference pipeline for deployment on milliwatt-level MCUs. Rather than focusing on new classification models, we systematically evaluate how other stages in the measurement pipeline affect the performance of the chosen model. Starting from MBNV1x0.25 as a baseline, we explore both handcrafted and algorithm-driven image preprocessing schemes, followed by a comprehensive assessment of four model compression techniques (i.e., post-training quantization (PTQ), quantization-aware training (QAT), pruning, and weight clustering) applied individually and in combination. All models are deployed on an STM32H7-based OpenMV board using Google Lite Runtime (LiteRT)^1^. The complete pipeline, from image capture to inference, is profiled in terms of latency, memory, energy, and effects on UAV flight time. This study provides a reproducible and practical reference for locating the accuracy–resource–energy trade-off in ultra-constrained UAV-based SHM applications.

The main contributions of this study are summarized as follows:

- **Optimized TinyML pipeline:** We present the first systematic Tiny Machine Learning study on crack classification that couples a greedy algorithm based preprocessing pipeline with 8-bit PTQ, QAT, pruning, and weight clustering (as well as their hybrids), raising the F1-score of a quantized MBNV1x0.25 model from 76% to 93.8%.
- **Milliwatt-level end-to-end deployment and measurement:** On an STM32H7-based OpenMV board the proposed pipeline attains 408.3 ms model inference time, 461.6 ms full-pipeline latency, a footprint of 2.9 MB RAM and 309 KB flash memory, and 623.16 mJ energy consumption per inference.
- **Quantified UAV flight-time analysis:** Using the DJI Mini 4 Pro as an example, our MCU-based solution shortens flight time by only 1.31 min (i.e., 4%).

The remainder of this paper has the following structure. In Section , the dataset used in this study is introduced. Section introduces the methodology including model training and validation, image preprocessing strategies, model compression and on-device integration. Section presents the obtained results and discussion. Finally, Section concludes the paper and outlines future work (Table 1).

## Dataset

**Figure 1:**
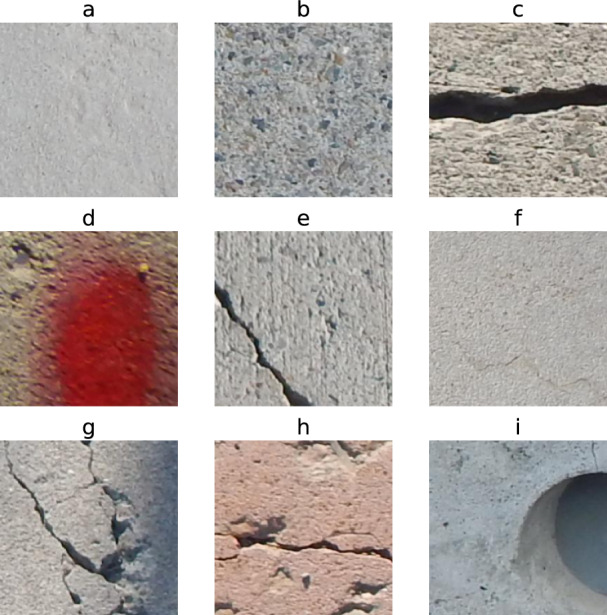
SDNET2018 images include (**a**), (**b**) & (**f**) fine cracks, (**c**), (**e**), (**g**) & (**h**) coarse cracks, (**d**) stains, (**i**) inclusions and voids^26^.

**Table 1:** SDNET2018 image dataset composition.

Image description	With crack	Without crack	Total
Reinforced Bridge deck	2025	11,595	13,620
Reinforced Wall	3851	14,287	18,138
Unreinforced Pavement	2608	21,726	54,334
Total	8484	47,608	56,092

In this study, the SDNET2018 dataset^26^ is used as the primary benchmark. SDNET2018 is a widely adopted dataset for AI-based concrete crack detection and classification, comprising over 56,000 images of concrete bridge decks, walls, and pavements, with and without cracks. The dataset includes various real-world disturbances such as shadows, surface roughness, edges, holes, scale variations, and background debris. Images were captured using a 16 MP Nikon camera at a working distance of approximately 500 mm, with a resolution of $4068 \times 3456$ pixels and illumination levels ranging from 1500 to 3000 lx. Following the original dataset protocol, each high-resolution image was divided into non-overlapping $256 \times 256$ pixel patches, each corresponding to a physical area of approximately $60 \times 60$ mm. To mitigate class imbalance, a subset of 16,800 patches was constructed, including 8,400 crack and 8,400 non-crack samples.

To reduce the risk of spatial correlation between training and testing data, the dataset split was performed at the level of the original high-resolution images rather than at the individual patch level. All patches extracted from the same parent image were assigned exclusively to either the training, validation, or test set. An 8:1:1 split ratio was adopted, resulting in 13,440 training samples, 1,680 validation samples, and 1,680 test samples. Finally, all patches were resized to $224 \times 224$ RGB images to match the input requirements of the pretrained backbone networks. Representative examples of the dataset are shown in Fig. 1.

## Methodology

**Figure 2:**
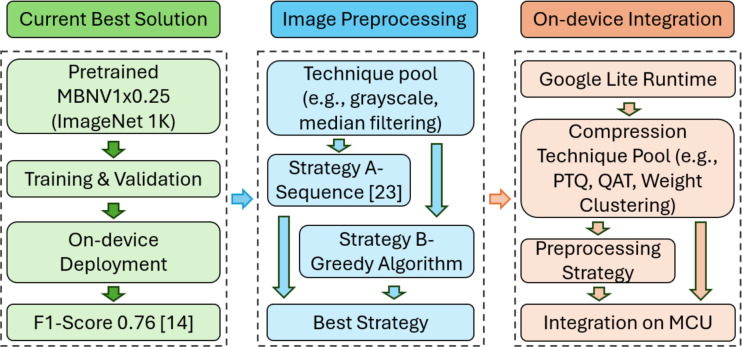
Overall methodology of this work, including model baseline, preprocessing optimization, and MCU deployment with compression.

The methodology in this paper is illustrated in Fig. 2, which is divided into two main stages: exploration of image preprocessing strategies, and on-device integration with model compression. First, a lightweight, pretrained MBNV1x0.25 model is trained with transfer learning for crack classification as baseline. Then different image preprocessing strategies are investigated to assess their effectiveness in enhancing performance. At the end, the best preprocessing strategy is integrated with different compression techniques, and is deployed on a low-power, resource-constrained MCU, where a comprehensive analysis of the system’s performance is conducted (Table 2).

### Model training and validation

**Table 2:** Architecture of MobileNetV1$\times$0.25 Used as the Baseline Model.

Layer Type	Kernel / Stride	Output Size	Channels
Input Image	–	$224 \times 224$	3
Conv2D	$3 \times 3$ / 2	$112 \times 112$	$32 \times 0.25 = 8$
Depthwise Conv	$3 \times 3$ / 1	$112 \times 112$	8
Pointwise Conv	$1 \times 1$ / 1	$112 \times 112$	$64 \times 0.25 = 16$
Depthwise Conv	$3 \times 3$ / 2	$56 \times 56$	16
Pointwise Conv	$1 \times 1$ / 1	$56 \times 56$	$128 \times 0.25 = 32$
Depthwise Conv	$3 \times 3$ / 1	$56 \times 56$	32
Pointwise Conv	$1 \times 1$ / 1	$56 \times 56$	32
Depthwise Conv	$3 \times 3$ / 2	$28 \times 28$	32
Pointwise Conv	$1 \times 1$ / 1	$28 \times 28$	$256 \times 0.25 = 64$
Depthwise Conv	$3 \times 3$ / 1	$28 \times 28$	64
Pointwise Conv	$1 \times 1$ / 1	$28 \times 28$	64
Depthwise Conv	$3 \times 3$ / 2	$14 \times 14$	64
Pointwise Conv	$1 \times 1$ / 1	$14 \times 14$	$512 \times 0.25 = 128$
Depthwise Conv $\times 5$	$3 \times 3$ / 1	$14 \times 14$	128
Pointwise Conv $\times 5$	$1 \times 1$ / 1	$14 \times 14$	128
Depthwise Conv	$3 \times 3$ / 2	$7 \times 7$	128
Pointwise Conv	$1 \times 1$ / 1	$7 \times 7$	$1024 \times 0.25 = 256$
Depthwise Conv	$3 \times 3$ / 1	$7 \times 7$	256
Pointwise Conv	$1 \times 1$ / 1	$7 \times 7$	256
Global Avg Pool	–	$1 \times 1$	256
Fully Connected	–	$1 \times 1$	$N_{class}$

In this work, we use MBNV1x0.25 (pretrained using ImageNet-1K) as the baseline model. MBNV1$\times$0.25 is adopted as the baseline model due to its well-established lightweight design and proven suitability for resource-constrained devices. MobileNetV1 employs depthwise separable convolutions, which decompose standard convolutions into depthwise and pointwise operations, significantly reducing both computational complexity and parameter count. The width multiplier of 0.25 further scales down the number of channels across all layers, leading to a compact model with a small memory footprint and low inference latency. These characteristics make MBNV1$\times$0.25 particularly suitable for deployment on MCUs, where strict constraints on flash memory, RAM, and power consumption must be satisfied. As a widely used TinyML backbone, it provides a reasonable and interpretable baseline for evaluating lightweight model performance under embedded deployment conditions. Also, in^14^, it was shown that MBNV1x0.25 provides a good trade-off between accuracy and resource consumption for SDNET2018. The model is fine-tuned on the SDNET2018 dataset, retraining all model parameters. The training environment used a Windows 10 64-bit operating system, an Intel®Core i9 12900 CPU, 32GB RAM, and a single RTX 3090 GPU with 24GB memory. We used the TensorFlow 2.8.0 framework, and model training is performed with a learning rate of 0.001 over 10 epochs.

For the evaluation of the model performance, the F1-score, precision, recall and accuracy are used as key metrics. They can be calculated according to

1 $$\begin{aligned} F1 = \frac{2 * Precision * Recall}{Precision + Recall} \end{aligned}$$

2 $$\begin{aligned} Precision = \frac{TP}{TP + FP} \end{aligned}$$

3 $$\begin{aligned} Recall = \frac{TP}{TP + FN} \end{aligned}$$

where TP, FP, and FN are the true positive, false positive, and false negative predictions, respectively.

### Image preprocessing strategies

The MBNV1x0.25 model was applied to preprocessed image datasets. The training and validation setup, as well as the evaluation metrics remain the same. The image preprocessing techniques used in this study include the following: (a) Grayscale conversion: transforms images from RGB to grayscale to reduce image complexity; (b) Median filtering: applies a median filter to remove noise while preserving edges; (c) Black Hat transformation: highlights darker regions on a lighter background, useful for crack detection; (d) White Hat transformation: emphasizes lighter regions on a darker background to enhance contrast; (e) Contrast adjustment: improves image contrast to enhance feature visibility; (f) Image denoising: reduces image noise to improve clarity and detection accuracy; and (g) Image binarization: converts images to binary form, isolating key features. All preprocessing techniques were initially implemented on a PC using OpenCV (version 4.10.0)^27^ to create the preprocessed datasets.

#### Strategy A - sequential method

In accordance with the preprocessing strategy proposed in^28^ for crack classification, we applied a series of image preprocessing steps. The image is first converted to grayscale using the weighted sum

4 $$\begin{aligned} Y = 0.299R + 0.587G + 0.114B \end{aligned}$$

where $Y$ is the grayscale intensity, and $R$, $G$, and $B$ are the red, green, and blue channel intensities, respectively. This transformation simplifies the image to a single channel, retaining important visual information while reducing computational complexity.

Next, contrast is adjusted with a contrast control $\alpha$ and a brightness control $\beta$. The adjusted image is obtained according to

5 $$\begin{aligned} \text {new\_image}(x, y) = \alpha \cdot \text {original\_image}(x, y) + \beta \end{aligned}$$

where $\alpha$ increases contrast, and $\beta$ is set to 0 to maintain brightness. A value of $\alpha = 1.5$ was chosen to moderately enhance contrast without excessive brightening, thus making structural details more discernible.

Denoising is employed with a bilateral filter applied with parameters $h = 10$, *template window size* = 7, and *search window size* = 21. This filter reduces noise while preserving edges, as it smooths regions with similar intensity values within a specified spatial neighborhood. The chosen parameters ensure effective noise reduction on textured surfaces while maintaining the clarity of crack edges.

After that, a median filter with a kernel size of 5 is applied to further smooth the image. This filter replaces each pixel value with the median value (calculated by bubble sort) of the surrounding pixels within a 5x5 neighborhood. This size was selected to balance noise removal with detail retention, as larger kernels could overly blur fine features such as crack edges.

Finally, binary thresholding with a threshold value of 241 is applied with

6 $$\begin{aligned} \text {dst}(x, y) = {\left\{ \begin{array}{ll} 255 & \text {if } \text {src}(x, y) > 241 \\ 0 & \text {otherwise} \end{array}\right. } \end{aligned}$$

This high threshold value isolates the brightest regions, which typically correspond to cracks or other prominent features against the concrete background, enhancing the final binary segmentation of the structural defects. To be noticed, all weight values were obtained based on experimental data, determined through manual inspection to achieve the best possible performance.

#### Strategy B - greedy algorithm-based method

The greedy algorithm^29^ is an approach that selects the best-performing choice at each step, aiming to achieve a globally best solution through a series of locally effective choices. The core idea of this algorithm is to make the most favorable choice at each decision point without backtracking or considering the overall structure, which generally results in lower time complexity and makes it suitable for specific optimization problems. Although the greedy algorithm does not guarantee a globally best-performing solution in all cases, its straightforward logic and low implementation cost make it advantageous for use in this study. The specific strategy for exploring combinations of image preprocessing techniques based on the greedy algorithm is as follows:

**Algorithm 1:**
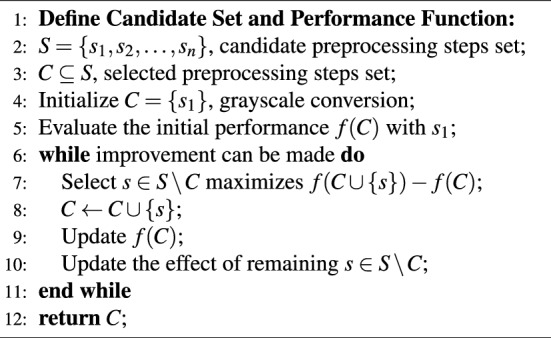
GreedyImagePreprocessingSelection()

This greedy strategy starts by applying grayscale conversion and evaluating the resulting model performance. In each iteration, it selects the preprocessing technique *s* from the remaining candidates that maximizes the performance improvement. This ensures each selected technique has the most significant positive impact on model performance. The chosen technique *s* is then added to the set *C*, and the current performance metric *f*(*C*) is updated. At the same time, the effects of the remaining candidate techniques on the new combination are recalculated, allowing the next iteration to continue selecting the effective step. Finally, when no preprocessing technique can improve performance, the algorithm terminates. The final output *C* represents the best combination of preprocessing steps.

### Model compression workflow and on-device integration

**Figure 3:**
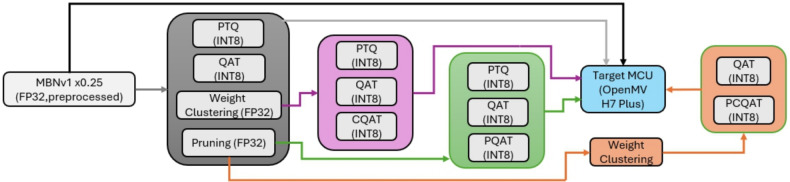
The model compression pipeline applies multiple strategies to a preprocessed MobileNetV1x0.25 model (FP32), including pruning, weight clustering, PTQ, and QAT. Different combinations-such as CQAT, PQAT, and PCQAT-are evaluated for deployment on the OpenMV H7 Plus MCU.

To enable efficient deployment of deep neural networks on resource constrained MCUs, this study investigates four mainstream model compression techniques and their compound variants: PTQ, QAT, Pruning, and Weight Clustering. All the techniques are part of the LiteRT framework. To ensure a fair comparison, all compression methods were evaluated under identical training and evaluation settings, including the same backbone architecture, dataset splits, training schedules, and deployment platforms. The only varying factor across experiments was the compression or quantization strategy.

- **PTQ** reduces model precision after training without modifying weights or retraining. In this study, we implement PTQ from FP32 to INT8.
- **QAT** simulates low-bit quantization during training to adapt model weights to quantized constraints. Similar to PTQ, the quantization is set to INT8.
- **Pruning (P)** removes redundant or low-magnitude weights or neurons from the network to reduce model size and computational cost while preserving performance. Half of the original weights are retained in this study.
- **Weight Clustering (WC)** groups similar weight values into shared centroids, enabling parameter compression through reduced numerical precision and improved weight reuse. The amount of group cluster are set to a value of eight.

In addition to standalone use, we also explore advanced combinations including:

- **CQAT** represents cluster preserving quantization aware training.
- **PQAT** means pruning preserving quantization aware training.
- **PCQAT** is sparsity and cluster preserving quantization aware training.

The complete compression and deployment pipeline is illustrated in Fig. 3, with 13 distinct processing routes (marked in different colors) all originating from the same baseline model. Specifically, we adopt the image-preprocessed MBNV1$\times$0.25 as the FP32 baseline throughout this study.

The evaluated variants include:

- **FP32 baseline**: MBNV1x0.25 without compression
- **PTQ**: MBNV1x0.25 with 8-bit post-training quantization
- **QAT**: MBNV1x0.25 with quantization-aware training
- **WC**: FP32 + weight clustering only
- **P**: FP32 + pruning only
- **WC + PTQ**: Clustering followed by PTQ
- **WC + QAT**: Clustering followed by QAT
- **WC + CQAT**: Clustering followed by clustered QAT
- **P + PTQ**: Pruning followed by PTQ
- **P + QAT**: Pruning followed by QAT
- **P + PQAT**: Pruning followed by pruning-aware QAT
- **P + WC + QAT**: Pruning and clustering followed by QAT
- **P + WC + PCQAT**: Pruning and clustering followed by pruning-clustering-aware QAT

These configurations allow a systematic analysis of compression effectiveness in terms of model size, memory footprint, and inference performance under the TinyML deployment paradigm.

**Table 3:** Technical specifications of OpenMV H7 Plus.

Parameter	OpenMV H7 Plus Development Board
MCU	STM32H743II
CPU Core	ARM Cortex M7
CPU Frequency	480MHz
RAM	32 MB SDRAM + 1 MB SRAM
Flash	32 MB external flash + 2 MB internal flash
Voltage	3.3V

To deploy the compressed models, we used the OpenMV H7 Plus as the target development board. This board is equipped with an STM32H743II ARM Cortex-M7 processor and integrates an OV5640 image sensor. Detailed specifications are provided in Table 3. Although the board features 32 MB of external SDRAM, the current firmware version (v4.5.6) supports a maximum of 4 MB for deep learning model memory. It should be noted that, while model pruning is applied as part of the compression pipeline to reduce parameter count, the current OpenMV deployment toolchain does not support sparse execution on MCUs. Consequently, pruned models are executed in a dense manner during inference, and the potential latency or energy benefits of sparsity cannot be fully realized on the target hardware. This limitation is explicitly considered when interpreting pruning-related results. The end-to-end workflow begins with model training in TensorFlow, followed by conversion to the LiteRT format. The compressed models are then deployed onto the OpenMV Cam H7 Plus using MicroPython, LiteRT, and the OpenMV IDE (version 4.4.7)^2^. Notably, the OpenMV Cam H7 Plus captures images at a resolution of $320 \times 240$ using the integrated OV5640 sensor. To match the input size required by the crack segmentation model, a centered $224 \times 224$ region is cropped from each frame and used as the model input.

Notably, PTQ and QAT were performed using the standard TensorFlow Lite (renamed as Google LiteRT) and TensorFlow Model Optimization Toolkit (TF-MOT) pipelines. For PTQ, the default TensorFlow Lite calibration procedure was adopted, where a randomly selected subset (10%) of training samples was used as the representative dataset to estimate activation ranges. No task-specific calibration heuristics were introduced. For QAT, fake quantization operators were inserted during training using the default TF-MOT configuration. The model was trained end-to-end with quantization simulated in the forward pass, and the final INT8 model was obtained via TensorFlow Lite conversion. Unless otherwise specified, all quantization parameters followed the default TensorFlow settings. All models were deployed on the OpenMV platform using its built-in TensorFlow Lite for Microcontroller runtime. Optimized MCU kernels provided by the default Google TFLite Micro backend were enabled, while no custom low-level kernel implementations (e.g., hand-tuned CMSIS-NN kernels) were introduced.

In terms of image preprocessing, the OpenMV library is employed to implement the required operations on-device. While OpenMV and OpenCV are distinct libraries, OpenMV being tailored for MCU environments and OpenCV for general-purpose computing, the preprocessing steps are designed to be conceptually and functionally consistent across both platforms. Minor numerical differences may arise due to implementation-specific details, data representations, and hardware constraints; however, all reported deployment results in this study are obtained using the on-device OpenMV preprocessing pipeline to ensure consistency with practical embedded execution.

### Robustness evaluation under UAV-like degradations

To better approximate UAV inspection conditions, we evaluate the robustness of the deployed model under common image degradations caused by platform motion and varying illumination. We focus exclusively on the best-performing deployed configuration of above results, and apply degradations *only at inference time* to the test set while keeping training unchanged. Let $\textbf{x}\in \mathbb {R}^{H\times W\times C}$ denote a clean input patch and $\mathcal {T}(\cdot )$ denote a degradation operator. The degraded input is

7 $$\begin{aligned} \tilde{\textbf{x}} = \mathcal {T}(\textbf{x}; \boldsymbol{\theta }), \end{aligned}$$

where $\boldsymbol{\theta }$ controls the degradation severity.

**Motion blur.** We model UAV-induced motion blur by convolving the image with a linear blur kernel $\textbf{k}_{L,\phi }$ of length *L* and direction $\phi$:

8 $$\begin{aligned} \tilde{\textbf{x}} = \textbf{x} * \textbf{k}_{L,\phi }. \end{aligned}$$

**Illumination change.** We emulate illumination variations using a gamma/brightness transform:

9 $$\begin{aligned} \tilde{\textbf{x}} = \textrm{clip}\big (\alpha \cdot \textbf{x}^{\gamma } + \beta ,\, 0, 1\big ), \end{aligned}$$

where $\alpha$ controls contrast/scale, $\beta$ controls brightness shift, and $\gamma$ models nonlinear exposure changes.

For each degradation type, we define multiple severity levels and report the mean and standard deviation of classification accuracy across repeated runs (or repeated degradation samplings). We additionally report the relative accuracy drop:

10 $$\begin{aligned} \Delta \textrm{Acc} = \textrm{Acc}_{\text {clean}} - \textrm{Acc}_{\text {degraded}}. \end{aligned}$$

## Results and discussion

This section presents the following aspects: (1) the performance evaluation of various image preprocessing strategies applied to the model before and after quantization; (2) the deployment accuracy, memory usage, and energy consumption of the best image preprocessing strategy with different model compression techniques integrated with TinyML on the MCU; and (3) estimation of impact on the UAV’s flight time.

### Image preprocessing strategy analysis

**Figure 4:**
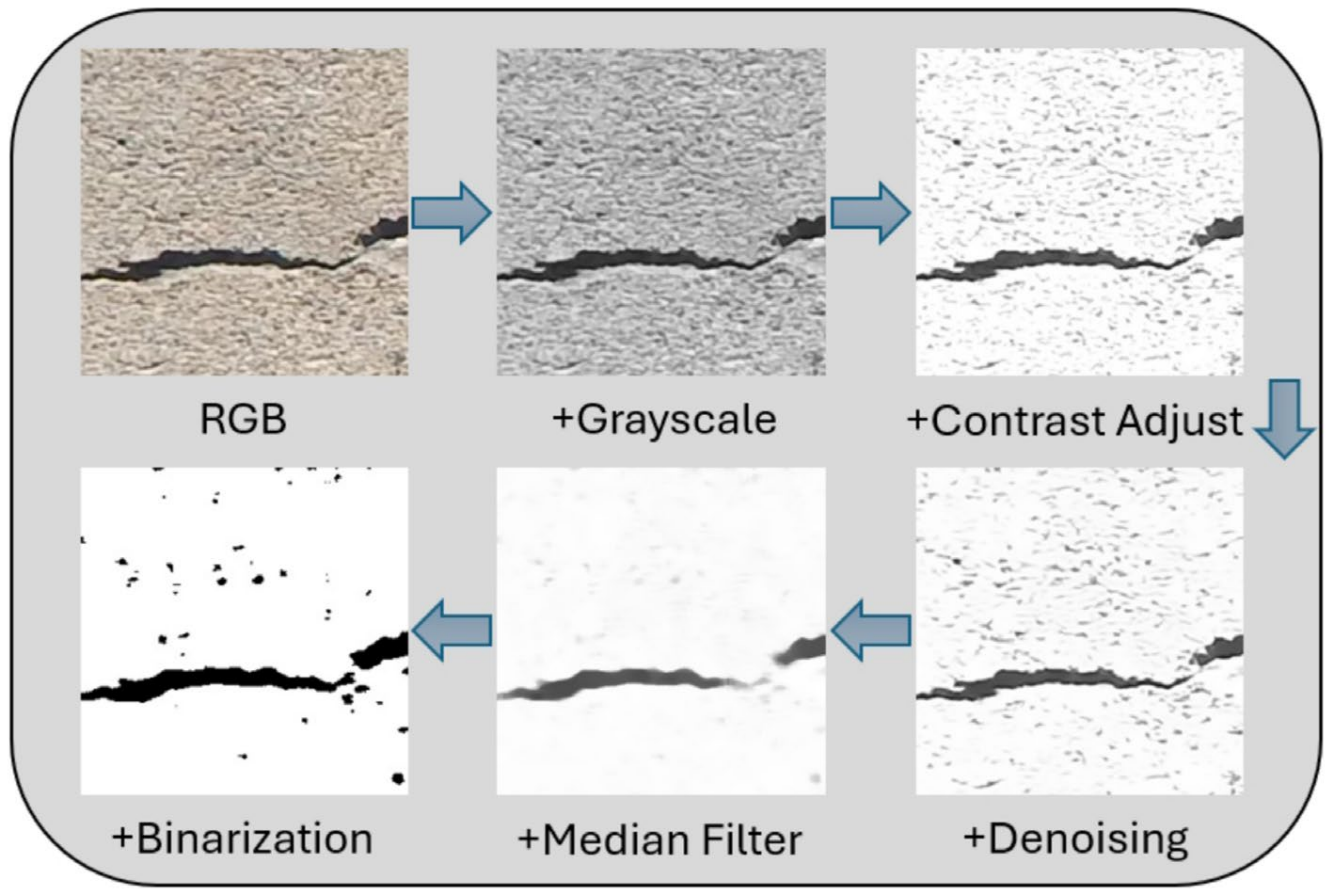
Image preprocessing sample with strategy A.

**Figure 5:**
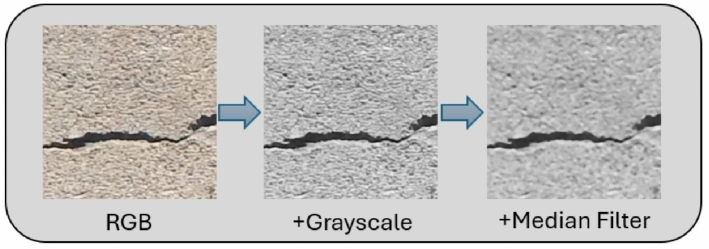
Image preprocessing sample with strategy B.

**Figure 6:**
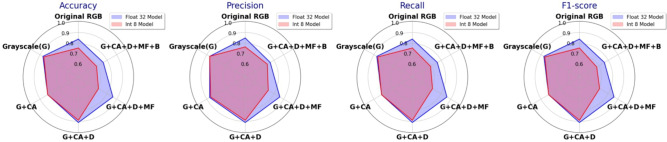
FP32 and INT8 MBNV1x0.25 performance with strategy A (CA-contrast adjustment, D-denoising, MF-median filtering, B-binary thresholding).

**Figure 7:**
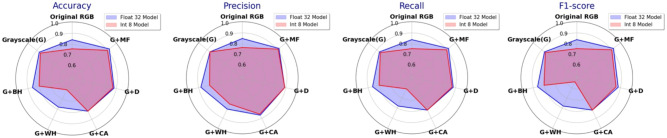
FP32 and INT8 MBNV1x0.25 performance with strategy B - step 1 (G + X) (WH-white hat, BH-black hat).

**Figure 8:**
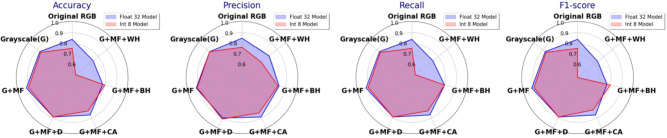
FP32 and INT8 MBNV1x0.25 performance with strategy B - step 2 (G+MF+X).

The image preprocessing stratergy A from^28^ with an image sample is presented in Fig. 4. The visualization of strategy B with the same image sample is shown in Fig. 5.

Figure 6 shows a notable performance difference between FP32 and INT8 (with PTQ) models. Converting the original RGB images to grayscale improves the performance of both formats, suggesting that grayscale conversion helps the model to focus on relevant structural features by reducing color noise. The addition of contrast adjustment produced mixed results: the F1-score of the FP32 model slightly decreases to 0.82, indicating an overemphasis on high-contrast areas, while the INT8 model’s F1-score remains stable at 0.82, demonstrating its adaptability to quantization effects. Adding denoising after grayscale and contrast adjustment significantly improves both performances, achieving the highest F1-score of 0.91 for the FP32 model and 0.89 for the INT8 model, as denoising effectively reduces noise and enhances the clarity of key features. However, performance decreases with the inclusion of median filtering, with the FP32 model’s F1-score dropping to 0.86 and the INT8 model’s to 0.71, possibly due to over-smoothing, which obscures structural details. Finally, binary thresholding leads to the most considerable performance drop (F1-score of 0.76 for the FP32 model and 0.68 for the Int8 model), likely because it removes important gradient information and overly simplified image features.

Overall, while strategy A aligns well with human perception for damage classification, MBNV1x0.25 shows limited performance in combination with this strategy. The best preprocessing combination was grayscale + contrast adjust + denoising, as it retains essential information and reduces noise, achieving a balanced F1-score of 0.89 in quantized deployments. Additional steps like median filtering and binary thresholding, although effective in^28^, reduce performance in crack detection, especially in low-precision models.

The performance of image preprocessing combinations selected by the greedy algorithm across different model precisions is shown in Fig. 7. On top of grayscale conversion, the addition of Black Hat and White Hat transformations produces varying results. Adding Black Hat raises the FP32 model’s F1-score to 0.86 but reduces the INT8 models score to 0.80. White Hat, on the other hand, leads to a significant performance drop, especially for the INT8 model, whose F1-score falls to 0.54, suggesting that this transformation may oversimplify image features, thus impairing model performance. Adding contrast adjustment and denoising improves model performance. Especially denoising, which achieves an F1-score of 0.88 for the FP32 model and 0.86 for the INT8 model, has significant benefits. Finally, the best performance is achieved by adding median filtering to the grayscale image, resulting in an F1-score of 0.92 for the FP32 model and 0.90 for the INT8 model, indicating that this preprocessing technique is highly effective in noise suppression and feature retention.

The performance of models after incrementally adding a third preprocessing techniques to the grayscale + median filter baseline using the greedy algorithm is presented in Fig. 8. After applying denoising, both models experience a slight decrease in F1-scores, indicating that denoising does not provide significant benefits on this baseline combination. Adding contrast adjustment further impacts performance, reducing F1-scores to 0.87 (FP32) and 0.82 (INT8), possibly due to unnecessary brightness enhancement in already clear features. Additional Black Hat and White Hat transformations cause substantial performance declines, with White Hat reducing the INT8 model’s F1-score to 0.50, suggesting that these transformations may remove critical structural information, making it difficult for the model to recognize fine details. Overall, the results indicate that the grayscale + median filter combination is the best preprocessing strategy according to the greedy algorithm and additional preprocessing steps do not improve performance and may even impair the model’s ability to recognize detailed features.

In summary, the best-performing preprocessing chain was identified using strategy B and consists of grayscale + median filtering. It outperforms strategy A explored in^28^, even when removing some degrading steps (i.e., mainting only grayscale + contrast adjustment + denoising). Through these preprocessing steps a high F1-scores 0.92 (FP32) and 0.90 (INT8) has been achieved, which motivates their integration in the crack classification pipeline.

### Model compression and on-device deployment

**Figure 9:**
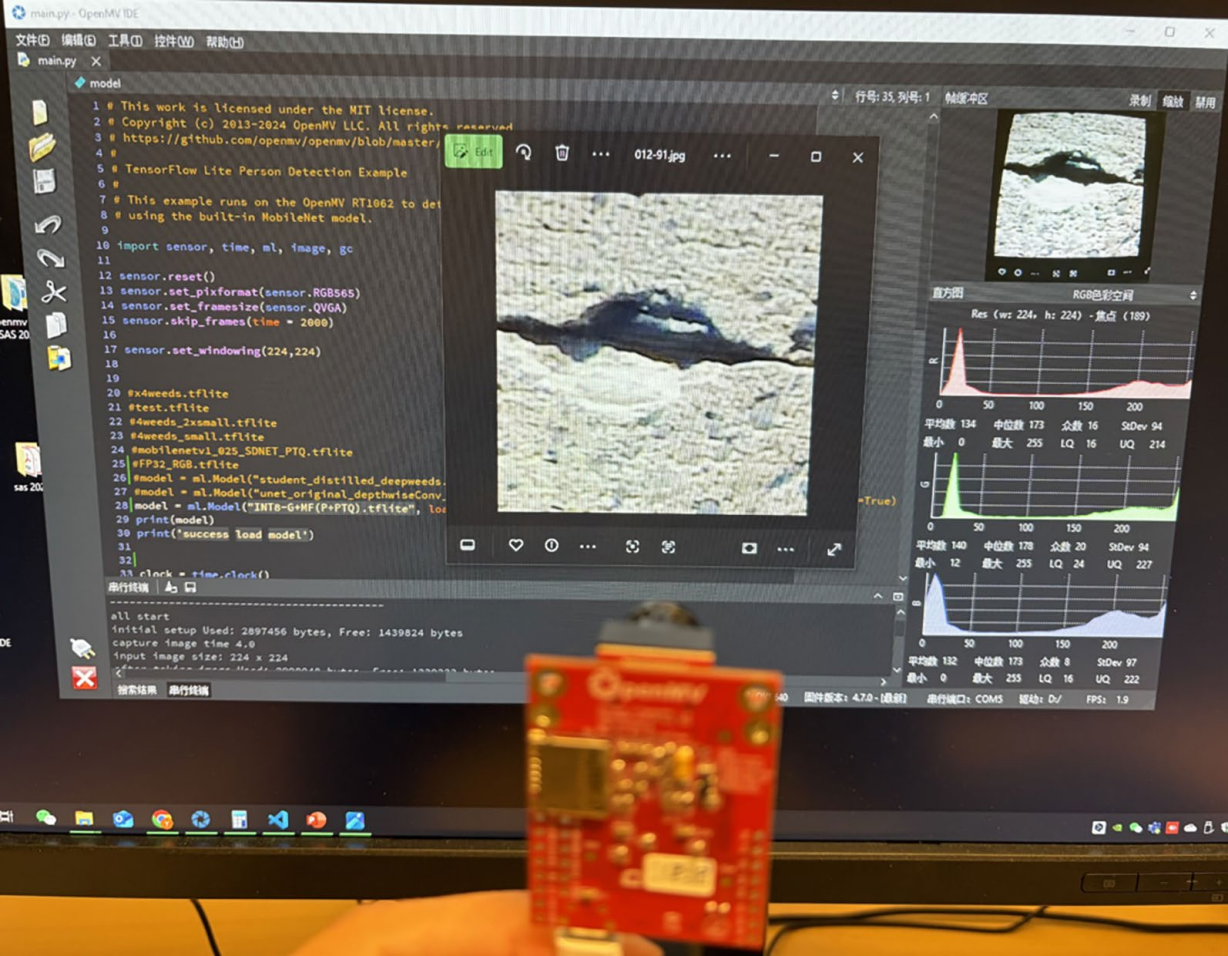
Real On-device Deployment (OpenMV H7 Plus) in Lab.

This section presents a comparison of model sizes after applying various compression techniques based on the FP32-G+MF baseline. It also reports the deployment results of the compressed models on the target device. The deployment evaluation includes real-device demonstrations shown in Fig. 9 and measurements of the crack classification module’s actual performance, including F1-score, flash and RAM usage, inference time, and energy consumption.

#### Model compression

**Table 4:** Model File Size Comparison under Different Compression Strategies.

Strategy	Zipped Model File Size (KB)
FP32-RGB (Original)	862
FP32-G+MF (P)	791
FP32-G+MF (WC)	217
FP32-G+MF (P+WC)	199
INT8-G+MF (P+WC+QAT)	209
**INT8-G+MF (P+WC+PCQAT)**	**125**
INT8-G+MF (PTQ)	305
INT8-G+MF (QAT)	307
INT8-G+MF (P+PTQ)	178
INT8-G+MF (P+QAT)	212
INT8-G+MF (P+PQAT)	177
INT8-G+MF (WC+PTQ)	145
INT8-G+MF (WC+QAT)	214
INT8-G+MF (WC+PQAT)	143

Table 4 summarizes the zipped model (by PC zip python function) sizes under different compression strategies. The uncompressed FP32-RGB baseline model occupies 862 KB, while the grayscale plus median filtering (G+MF) variant with pruning (P) reduces the size modestly to 791 KB. More substantial compression is achieved with weight clustering (WC), shrinking the model to 217 KB. The combination of pruning and weight clustering (P+WC) further reduces the size to 199 KB.

Among all strategies, the smallest model with only 125 KB is obtained using the INT8-G+MF (P+WC+PCQAT) approach, which combines pruning, weight clustering, and partially constrained quantization-aware training (PCQAT). This represents an 85.5% reduction compared to the FP32-RGB baseline, and a 57.3% reduction compared to standard INT8 post-training quantization (PTQ).

Comparing quantization strategies alone, INT8 PTQ and QAT models are relatively large (305 KB and 307 KB, respectively), suggesting that quantization without further structural compression is insufficient for extreme memory-constrained applications. By integrating pruning or clustering, models such as INT8-G+MF (P+PTQ) and (WC+PTQ) can achieve sizes of 178 KB and 145 KB, respectively, indicating the synergy between structural and quantization-based compression techniques.

Overall, combining pruning, weight clustering, and QAT or PCQAT leads to the most compact and efficient model representations, making them especially suitable for deployment on ultra-low-power MCUs with strict memory constraints.

#### On-device deployment

The zipped model sizes reported in Table 4 do not fully reflect the actual flash memory consumption observed during on-device deployment, as shown in Table 5. This discrepancy arises because the LiteRT files deployed using the LiteRT toolchain are not compressed using any zip functionality. Therefore, while Table 4 offers a useful reference for relative size reduction across strategies, Table 5 provides a more accurate estimate of the memory footprint in real deployment scenarios.

**Table 5:** Compressed and Deployed Model Performances on OpenMV H7 Plus (model inference only & whole system). Flash, RAM in Bytes, Time in ms, Energy in mJ.

Compression techniques with MBNV1x0.25	F1-score	Flash (whole system)	RAM (whole system)	Inference Time (model only)	Inference Time (whole system)	Energy Consumption per Inference (whole system)
FP32-Original	0.842± 0.023	862,375	3,642,224	1,297.8 ± 3.8	1,300.3 ± 3.9	1,755.405
FP32-G	0.863± 0.015	862,375	3,642,224	1,301.5 ± 3.9	1,304.4 ± 4.0	1,760.94
FP32-G+MF	0.921± 0.033	862,375	3,642,224	1,352.7 ± 4.5	1,355.6 ± 4.1	1,830.06
FP32-G+MF (P)	0.904± 0.038	862,747	3,642,160	1,301.3 ± 4.5	1,354.2 ± 4.1	1,828.17
FP32-G+MF (WC)	0.866± 0.027	862,743	3,642,176	1300.2 ± 4.2	1353.2 ± 4.0	1,826.82
FP32-G+MF (P+WC)	0.839± 0.019	862,743	3,642,176	1304.2 ± 3.8	1357.7 ± 3.9	1,832.895
INT8-G+MF (PTQ)	0.904± 0.031	**307,051**	**2,796,752**	**398.5 ± 5.8**	**452.2 ± 6.2**	**610.47**
INT8-G+MF (QAT)	0.906± 0.034	308,635	2,796,832	408.2 ± 4.8	461.4 ± 5.4	622.89
INT8-G+MF (P+PTQ)	0.888± 0.015	**307,051**	**2,796,752**	401.8 ± 8.8	455.8 ± 9.5	615.33
INT8-G+MF (P+QAT)	**0.938± 0.022**	308,635	2,898,304	408.3 ± 8.4	461.6 ± 8.8	623.16
INT8-G+MF (P+PQAT)	0.695± 0.041	308,643	2,898,304	408.4 ± 7.5	461.8 ± 7.8	623.43
INT8-G+MF (WC+PTQ)	0.849± 0.030	**307,051**	**2,796,752**	402.5 ± 6.3	456.4 ± 6.8	616.14
INT8-G+MF (WC+QAT)	0.919± 0.027	308,643	2,898,304	410.2 ± 7.2	463.8 ± 7.7	626.13
INT8-G+MF (WC+CQAT)	0.501± 0.029	308,643	2,898,304	410.5 ± 9.3	463.9 ± 9.9	626.265
INT8-G+MF (P+WC+QAT)	0.835± 0.034	308,635	2,898,304	414.2 ± 14.5	468.1 ± 15.4	631.935
INT8-G+MF (P+WC+PCQAT)	0.845± 0.027	308,643	2,898,304	414.5 ± 14.8	468.2 ± 15.5	632.07

Table 5 reports deployment-level performance metrics under real on-device execution conditions, where F1-score is averaged over ten independent trainings, and all other values are averaged over ten independent runs and presented as mean ± standard deviation. The metrics include flash and RAM usage (whole system), inference time (both model-only and full pipeline), and the energy consumption per inference cycle.

In comparison to the original FP32-RGB model (F1-score: 0.842), converting the input to grayscale (FP32-G) slightly improves accuracy to 0.863, and further gains are observed when median filtering is introduced (FP32-G+MF: 0.921). This validates the effectiveness of input preprocessing for enhancing model robustness without architectural changes. In addition, this demonstrates that OpenCV an OpenMV libraries achieve similar results.

Among the FP32 variants, pruning (P) and weight clustering (WC) individually reduce F1-score slightly, but remain competitive with 0.904 and 0.866, respectively. However, their combination (P+WC) degrades performance to 0.839, suggesting a potential compounding effect of aggressive structural compression. Interestingly, all FP32 variants occupy nearly identical flash and RAM footprints ( 862 KB and 3.6 MB), indicating that structural modifications alone do not reduce memory load without quantization. This discrepancy from the compression results in Table 4 is mainly due to the lack of support for pruning and weight clustering in the current deployment toolchain.

Quantization yields substantial memory and latency benefits. The INT8-G+MF (PTQ) and (QAT) models reduce flash usage by nearly 64% (to  307 KB), cut RAM usage by 23%, and accelerate inference time by approximately 65% (from  1355 ms to  452 ms full-pipeline latency), while maintaining comparable F1-scores (0.904 and 0.906). These results highlight quantization as a highly effective compression strategy for deployment on resource-constrained MCUs.

Combining pruning or clustering with quantization yields mixed results. The best-performing model in terms of accuracy is INT8-G+MF (P+QAT), achieving an F1-score of 0.938 with minimal latency increase (461.6 ms) and only marginal memory cost (308 KB flash, 2.9 MB RAM). Interestingly, we observe that the combination of pruning and QAT (P+QAT) results in higher RAM usage compared to QAT alone. This counterintuitive outcome stems from the deployment toolchain’s lack of support for sparse model representations. Although pruning reduces the number of non-zero parameters, the model is still stored and executed as a dense network, and additional overhead from tensor reshaping or internal memory alignment may further increase runtime memory requirements. This suggests that QAT can be used to further improve accuracy based on the floating-point benchmark model. On the other hand, PQAT and CQAT variants (e.g., INT8-G+MF (P+PQAT), F1-score: 0.695; and (WC+CQAT), F1-score: 0.501) suffer from significant performance drops, likely due to training instability or overly aggressive constraints.

Notably, the most memory-efficient model, INT8-G+MF (PTQ), consumes only 610.47 mJ per inference, compared to 1830.06 mJ for the FP32-G+MF baseline, representing a 66.7% reduction in energy. Even the slightly larger INT8-G+MF (P+QAT) variant retains low energy usage (623.16 mJ) while achieving the highest accuracy overall. It should be also noted that the reported performance improvements are the result of multiple interacting design choices, including preprocessing strategy, model compression, and retraining. While these components are evaluated in combination to reflect realistic deployment pipelines, their marginal contributions can be interpreted by examining controlled comparisons across experiments. Specifically, comparisons between sequential and greedy preprocessing under identical model and quantization settings highlight the impact of preprocessing design. Similarly, comparisons across PTQ and QAT configurations under fixed preprocessing isolate the effect of compression and retraining. Based on these results, preprocessing primarily improves robustness to background noise and texture variations, while quantization-aware retraining mainly preserves accuracy under aggressive INT8 deployment. Their combination yields the most stable accuracy–efficiency trade-off.

In summary, quantization significantly improves memory efficiency and inference speed while maintaining or even improving accuracy when combined with appropriate training strategies. Among all candidates, INT8-G+MF (P+QAT) achieves the best balance between classification performance (F1-score: 0.938), latency (461.6 ms), memory usage (308 KB flash), and energy efficiency (623.16 mJ), making it the most suitable model for deployment in low-power edge-based SHM applications.

### Error analysis of the best framework

**Table 6:** Confusion matrix of the INT8-G+MF (P+QAT) model on the test set (1680 samples).

Ground Truth	Predicted Crack	Predicted Non-crack
**Crack**	794 (TP)	46 (FN)
**Non-crack**	59 (FP)	781 (TN)

**Figure 10:**
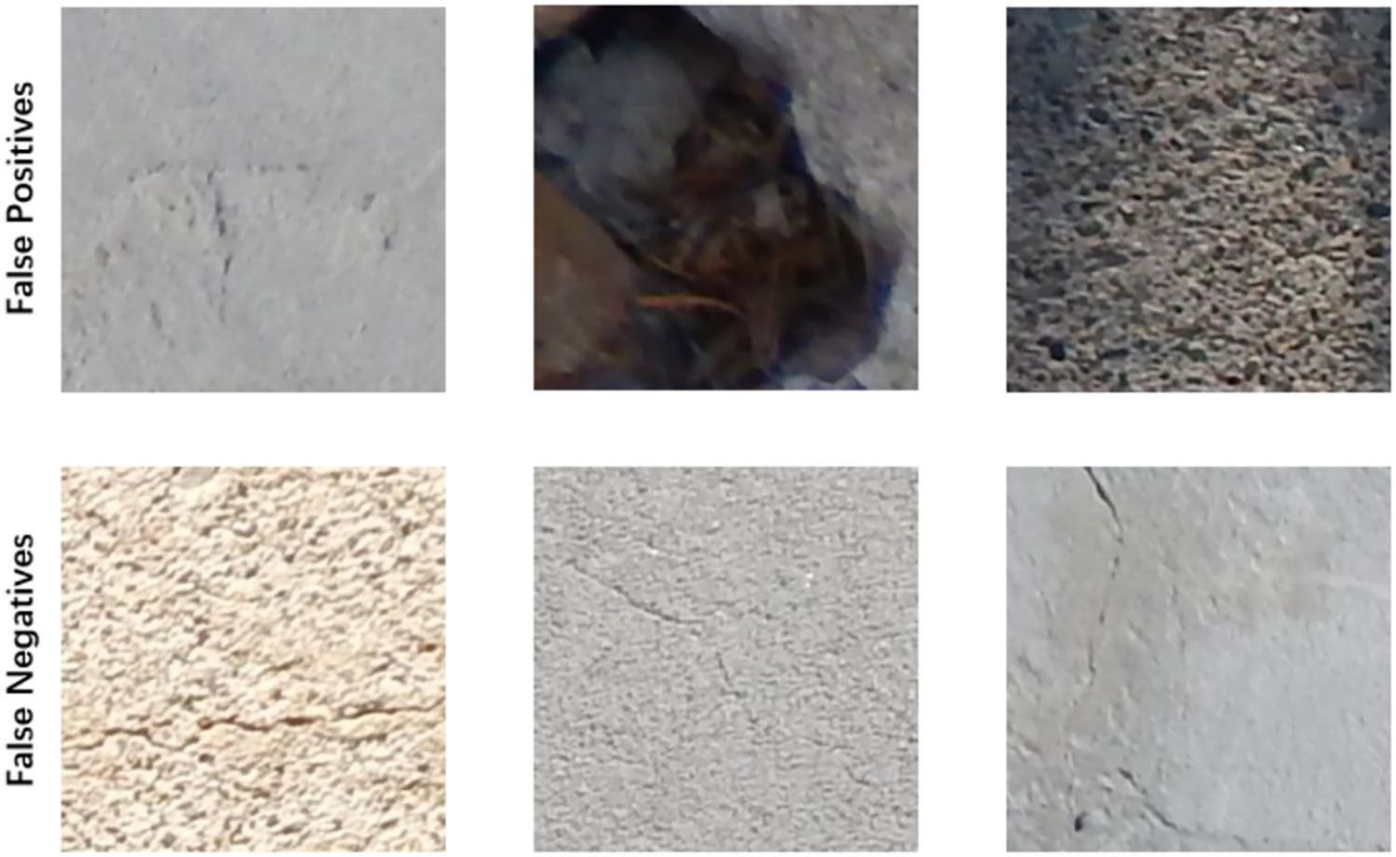
Typical False Positive / False Negative Examples.

As shown in Table 6 and Figure 10, most misclassifications arise from visually ambiguous patterns. False positives are mainly caused by surface seams, stains, or strong shadows that resemble crack-like structures, while false negatives typically occur for fine or low-contrast cracks embedded in textured backgrounds.

### Robustness analysis results under UAV-like degradations

**Table 7:** Robustness check of the deployed INT8-G+MF (P+QAT) model under UAV-like degradations. Clean baseline accuracy: $0.938 \pm 0.022$. $\Delta$Acc denotes the absolute accuracy drop from the clean baseline.

Degradation	Severity setting	Accuracy	$\Delta$Acc
**Clean**	–	$0.938 \pm 0.022$	0.000
**Motion blur**	Mild ($L{=}5$ px)	$0.915 \pm 0.025$	0.023
	Moderate ($L{=}9$ px)	$0.882 \pm 0.030$	0.056
	Severe ($L{=}13$ px)	$0.831 \pm 0.034$	0.107
**Illumination change**	Mild ($\gamma {=}0.9, \beta {=}0.05$)	$0.927 \pm 0.023$	0.011
	Moderate ($\gamma {=}0.8, \beta {=}0.10$)	$0.906 \pm 0.026$	0.032
	Severe ($\gamma {=}0.7, \beta {=}0.15$)	$0.879 \pm 0.030$	0.059

Table 7 reports the robustness of the deployed INT8-G+MF (P+QAT) model under representative UAV-like degradations, with a clean-test baseline accuracy of $0.938 \pm 0.022$. Notably, all degradations are applied on-the-fly at inference time without retraining. Overall, the model exhibits a consistent and monotonic performance degradation as the severity of each corruption increases, indicating stable and predictable behavior under adverse imaging conditions.

Among the evaluated factors, motion blur has the most pronounced impact on classification performance. Even under mild blur ($L=5$ px), the accuracy drops by 2.3 percentage points, and the degradation becomes substantial at higher blur levels, reaching a maximum decrease of 10.7 percentage points under severe blur. This sensitivity is expected, as motion blur directly suppresses high-frequency edge and texture cues that are critical for crack discrimination, particularly in patch-level classifiers operating on limited spatial context. The observed trend highlights motion-induced artifacts as a key challenge for UAV-based inspection, especially during fast flight or under wind disturbances.

In contrast, the model demonstrates higher robustness to illumination variations. Mild and moderate illumination changes result in relatively small accuracy drops (1.1 and 3.2 percentage points, respectively), suggesting that the combination of grayscale conversion, morphological filtering, and quantization-aware training contributes to a degree of illumination invariance. Although performance degradation becomes more noticeable under severe illumination shifts, the accuracy remains above 0.87, indicating that the model retains reasonable discriminative capability even under challenging lighting conditions.

It is also worth noting that the standard deviation of accuracy increases slightly with degradation severity, reflecting higher prediction uncertainty as image quality deteriorates. Nevertheless, the overall variance remains moderate, suggesting that the quantized INT8 deployment maintains stable inference behavior across repeated runs.

Taken together, these results suggest that the proposed INT8-G+MF (P+QAT) model is reasonably robust to common UAV-induced visual degradations, with motion blur being the dominant limiting factor. This observation motivates future work on integrating blur-aware data augmentation, lightweight deblurring modules, or flight-aware adaptive inference strategies to further enhance robustness in real-world UAV deployments.

### Evaluation under class imbalance

To reflect practical inspection scenarios where non-crack samples dominate, we further evaluate the deployed INT8-G+MF (P+QAT) model under artificially imbalanced test sets. Class imbalance is introduced by subsampling crack patches while keeping the non-crack samples unchanged. Precision, recall, and F1-score are reported to assess practical decision behavior.

**Table 8:** Performance of the deployed INT8-G+MF (P+QAT) model under different class imbalance ratios. Precision (P), Recall (R), and F1-score (F1) are reported for the crack class.

Class ratio (Crack:Non-crack)	Precision	Recall	F1-score
1:1 (Balanced)	$0.931 \pm 0.024$	$0.945 \pm 0.021$	$0.938 \pm 0.022$
1:5	$0.956 \pm 0.020$	$0.901 \pm 0.028$	$0.928 \pm 0.023$
1:10	$0.972 \pm 0.018$	$0.864 \pm 0.031$	$0.915 \pm 0.026$

Table 8 summarizes the model behavior under increasing class imbalance. As the proportion of non-crack samples increases, precision consistently improves, indicating a reduced false-alarm rate, while recall gradually decreases due to the more conservative decision boundary. Despite this trade-off, the F1-score remains relatively stable, suggesting that the deployed INT8-G+MF (P+QAT) model maintains balanced performance even under highly imbalanced conditions. This behavior is desirable for practical UAV inspection scenarios, where minimizing false positives is often prioritized to reduce unnecessary follow-up inspections.

### Impact on UAVs’ flight time

For the evaluation of UAV flight time, it is assumed that the primary function of the UAV is flight, while a dedicated embedded system performs crack classification tasks. In this study, the DJI Mini 4 Pro UAV is selected as a reference platform, featuring a battery capacity of 18.96 Wh, a nominal flight duration of 34 minutes, and an average propulsion power consumption of 33.46 W^30^. Under this illustrative scenario, the TinyML module is powered directly by the UAV battery.

To simplify the analysis, the impact of additional payload weight and thermal effects introduced by onboard computation are not explicitly modeled. Instead, the energy consumption analysis focuses on the electrical power required for inference operations, with the goal of providing a first-order estimation of flight-time impact under lightweight embedded deployment assumptions.

As an illustrative example, the INT8-G+MF (P+QAT) MobileNetV1$\times$0.25 configuration is considered. Based on the technical specifications of the target MCU, including operating voltage, clock frequency, and current consumption, the power consumption during active inference is estimated to be approximately 1.35 W. Assuming continuous inference throughout the entire flight duration, the total system power consumption increases to 34.81 W, resulting in an estimated flight time of 32.69 minutes. During this period, approximately 3,147 inference operations can be executed.

In a more realistic deployment scenario, inference is expected to be performed intermittently rather than continuously. For example, assuming an inference interval of 3 seconds, accounting for image acquisition, brief stabilization, and computation, the TinyML module is active for roughly one third of the mission time. Under this duty-cycled operation, the average power consumption of the crack inspection module is reduced to approximately 0.45 W, yielding a total system power consumption of 33.91 W. Consequently, the estimated flight time is reduced by only about 1.4%, from 34.00 minutes to approximately 33.52 minutes.

In addition to power consumption, payload weight is an important factor influencing UAV endurance. For reference, the NVIDIA Jetson Xavier NX module has a mass of approximately 182 g^3^, whereas the OpenMV H7 Plus board weighs only about 17 g^4^. This substantial reduction in payload mass further supports the suitability of lightweight TinyML hardware for UAV-based inspection, as reduced payload weight generally translates to improved flight endurance, especially for small UAV platforms.

It should be emphasized that this analysis is intended to provide an indicative comparison rather than a comprehensive sensitivity study. A more detailed investigation accounting for payload-induced aerodynamic effects, thermal dissipation, and mission-specific duty cycles will be considered in future work.

## Conclusions and future work

This study systematically optimizes the end-to-end TinyML inference pipeline for autonomous crack classification on low-power, resource constrained MCUs. Instead of proposing a new model architecture, we focus on evaluating how the choice of image preprocessing and model compression techniques affects final model performance. Using MBNV1x0.25 as a baseline, we compare a handcrafted grayscale–median filtering preprocessing pipeline with an algorithm-driven approach, and assess the impact of four mainstream compression techniques, covering PTQ, QAT, pruning, and weight clustering–both individually and in combination.

Our experimental results demonstrate that careful selection of preprocessing and compression strategies can significantly enhance both accuracy and efficiency. For instance, grayscale and median filtering alone improve the baseline F1-score from 0.842 to 0.921, while the combined INT8-G+MF (P+QAT) model achieves an F1-score of 0.938. The latter solution also reduces energy consumption by over 66%, and cuts flash usage by more than 60% compared to the FP32 baseline. Moreover, if pruning is supported by the deployment toolchain in the future, the size of the INT8-G+MF (P+QAT) model could be further reduced from 300 KB to 212 KB without any additional modifications, while inference time would also decrease accordingly. All models were deployed and profiled on the STM32H7-based OpenMV platform, offering reproducible performance metrics on memory, latency, and per-inference energy consumption. These findings highlight the importance of jointly optimizing preprocessing and compression to reach the accuracy-efficiency trade-off on constrained edge devices.

Although SDNET2018 is a widely used benchmark, subsampling the dataset to 16,800 images may reduce exposure to rare or fine-grained crack patterns. While the selected subset preserves class balance and overall visual diversity, extremely fine or ambiguous cracks may be underrepresented, which could potentially affect generalization across different crack scales and morphologies. Addressing such limitations is therefore an important direction for future work. In addition, the present study focuses exclusively on binary crack classification. While the proposed greedy preprocessing selection effectively enhances discriminative texture cues for classification, such preprocessing strategies may introduce biases that are not directly transferable to more complex tasks, such as pixel-level crack segmentation or multi-class damage classification, where spatial continuity, boundary preservation, and inter-class ambiguity play a more critical role. As a result, the current pipeline should be interpreted as classification-oriented, and its direct applicability to segmentation or multi-damage scenarios is not guaranteed. Future work will therefore extend this study toward more practical, robust, and portable deployment scenarios. First, beyond binary classification, TinyML-based crack segmentation and lightweight multi-class damage recognition will be explored to assess how preprocessing choices and model compression strategies generalize to tasks requiring finer spatial and semantic resolution. Second, while the current implementation relies on MicroPython and the OpenMV ecosystem for rapid prototyping, future efforts will focus on migrating the proposed pipeline to more widely adopted MCU software stacks, such as bare-metal C/C++, RTOS-based systems, and mainstream frameworks including ESP-IDF, RT-Thread, and Arduino-compatible cores, to improve portability across heterogeneous embedded platforms. Third, system-level integration with UAV platforms will be investigated, including communication interfaces, power management, and interaction with flight controllers, in order to ensure robust operation under realistic flight and environmental constraints. Finally, adaptive optimization pipelines, leveraging techniques such as hardware-aware neural architecture search and adaptive quantization, will be explored to balance accuracy, latency, and energy consumption across different embedded platforms. Together, these directions aim to advance the proposed approach toward a deployable TinyML-based SHM solution suitable for real-world UAV-assisted inspection workflows.

## Data Availability

The datasets used and/or analysed during the current study are available from https://www.kaggle.com/datasets/aniruddhsharma/structural-defects-network-concrete-crack-images.

## Footnotes

1 https://ai.google.dev/edge/litert

2 https://openmv.io/

3 http://www.plink-ai.com/en/product/nx16g-7f4.html

4 https://openmv.io/products/openmv-cam-h7-plus?srsltid=AfmBOoqMTFQNAIIrOMFJiMs9NSqZY1sVT72RuB5edL0Ed5Ht9OGqj3ZV
